# Assessing the comparative effects of interventions in COPD: a tutorial on network meta-analysis for clinicians

**DOI:** 10.1186/s12931-024-03056-x

**Published:** 2024-12-21

**Authors:** Katrin Haeussler, Afisi S. Ismaila, Mia Malmenäs, Stephen G. Noorduyn, Nathan Green, Chris Compton, Lehana Thabane, Claus F. Vogelmeier, David M. G. Halpin

**Affiliations:** 1 ICON Health Economics, ICON Plc, Langen, Germany; 2 Value Evidence and Outcomes, GSK, Collegeville, PA USA; 3 Department of Health Research Methods, Evidence and Impact, McMaster University, Hamilton, ON Canada; 4 ICON Health Economics, ICON Plc, Stockholm, Sweden; 5 Value Evidence and Outcomes, R&D Global Medical, GSK, Mississauga, ON Canada; 6 Department of Statistical Science, University College London, London, UK; 7 Global Medical, GSK, Brentford, UK; 8 St Joseph’s Healthcare Hamilton, Hamilton, ON Canada; 9 Department of Medicine, Pulmonary and Critical Care Medicine, Philipps-Universität Marburg, Member of the German Center for Lung Research (DZL), Marburg, Germany; 10 German Center for Lung Research (DZL), Marburg, Germany; 11 University of Exeter Medical School, College of Medicine and Health, University of Exeter, Exeter, UK

**Keywords:** Bayesian, Bucher ITC, Chronic obstructive pulmonary disease, Frequentist, GRADE, Head-to-head comparison, Indirect treatment comparison, Network meta-analysis, Randomized controlled trials, Single-inhaler triple therapy

## Abstract

To optimize patient outcomes, healthcare decisions should be based on the most up-to-date high-quality evidence. Randomized controlled trials (RCTs) are vital for demonstrating the efficacy of interventions; however, information on how an intervention compares to already available treatments and/or fits into treatment algorithms is sometimes limited. Although different therapeutic classes are available for the treatment of chronic obstructive pulmonary disease (COPD), assessing the relative efficacy of these treatments is challenging. Synthesizing evidence from multiple RCTs via meta-analysis can help provide a comprehensive assessment of all available evidence and a “global summary” of findings. Pairwise meta-analysis is a well-established method that can be used if two treatments have previously been examined in head-to-head clinical trials. However, for some comparisons, no head-to-head studies are available, for example the efficacy of single-inhaler triple therapies for the treatment of COPD. In such cases, network meta-analysis (NMA) can be used, to indirectly compare treatments by assessing their effects relative to a common comparator using data from multiple studies. However, incorrect choice or application of methods can hinder interpretation of findings or lead to invalid summary estimates. As such, the use of the GRADE reporting framework is an essential step to assess the certainty of the evidence. With an increasing reliance on NMAs to inform clinical decisions, it is now particularly important that healthcare professionals understand the appropriate usage of different methods of NMA and critically appraise published evidence when informing their clinical decisions. This review provides an overview of NMA as a method for evidence synthesis within the field of COPD pharmacotherapy. We discuss key considerations when conducting an NMA and interpreting NMA outputs, and provide guidance on the most appropriate methodology for the data available and potential implications of the incorrect application of methods. We conclude with a simple illustrative example of NMA methodologies using simulated data, demonstrating that when applied correctly, the outcome of the analysis should be similar regardless of the methodology chosen.

**Supplementary Information:**

The online version contains supplementary material available at 10.1186/s12931-024-03056-x.

## Plain Language Summary

There are several different treatments available for chronic obstructive pulmonary disease (COPD). Finding out which of these treatments is the most effective is difficult, especially if conflicting results from clinical trials have been reported, or if treatments have never been directly compared to each other. Meta-analysis allows the results from multiple studies to be combined together to give a single summary of findings. This can be useful in cases where previous trials have shown contradictory findings. However, this method can only be used if there is more than one study looking at the same two treatments (e.g., several studies that compared treatment A to treatment B). For treatments that have never been compared in clinical trials, network meta-analysis (NMA) can be used. This method allows several treatments to be compared at the same time using the results from trials comparing different treatments. This method creates ‘indirect evidence’. Indirect evidence refers to cases where two treatments have never been directly compared to each other in a clinical study, but both have been separately compared to a common treatment (e.g., treatment A and treatment C have never been directly compared to each other, but both have been separately compared to treatment B in a clinical study). NMA can be carried out using different methods. However, if the correct method is not chosen, this can lead to inaccurate results. It is becoming more common for NMA findings to be used to help make clinical decisions. Therefore, it is important that healthcare professionals are able to assess the results of published NMAs, including the methods used, to find the most appropriate results to support their clinical decisions. This tutorial provides an overview of different NMA methods, with a focus on the use of these methods within the context of COPD treatments. We also present an example where we use various NMA methods on the same data set to show that different methods should lead to similar results if the methods are used correctly.

**Supplementary Information:**

The online version contains supplementary material available at 10.1186/s12931-024-03056-x.

## Introduction

To optimize patient outcomes in chronic obstructive pulmonary disease (COPD), it is important that decisions on funding and reimbursement made by health technology assessment (HTA) bodies and payers are based on a thorough appraisal of the evidence for efficacy of treatments [1, 2]. It is also important that decisions made by healthcare professionals (HCPs) and management recommendations in national and international guidelines are based on the most up-to-date and highest-quality evidence available [3–7].

Randomized controlled trials (RCTs) demonstrate the efficacy of therapies, but often these studies do not compare all available treatments or provide information on how individual treatments fit into treatment algorithms [8]. RCTs usually compare the intervention of interest to an established treatment and/or placebo and are seldom replicated. RCTs examining the same treatments can also sometimes result in contradictory conclusions—this can be due to different trial designs or populations, but also as a result of random variation [9, 10]. Synthesizing evidence from multiple RCTs provides a balanced and comprehensive assessment of all available evidence on a given topic, as well as a “global summary” of findings. This is a fundamental way in which HTA bodies, payers, providers, and those developing clinical management guidelines make informed decisions [1, 3, 4, 11].

Various methods of evidence synthesis can be used in the development of management recommendations and HTA appraisals/reimbursement decisions, and HTAs and payers often have set preferred approaches. For example, the National Institute for Health and Care Excellence (NICE) Technical Support Documents (TSDs) on evidence synthesis make recommendations for preparing, reviewing, and appraising submissions to NICE [12]. However, the TSDs do not attempt to recommend the form that the analysis must take or the methods to be used. Any methods fulfilling the required properties are valid; the appropriateness of the approaches often depends upon the data available [13]. However, methodological problems can hinder interpretation of findings or lead to invalid summary estimates [14–17]. Such problems include inappropriate searching and selection of relevant trials for inclusion in analyses [18, 19], lack of publication bias assessment or evidence appraisal [20, 21], poor reporting of methodology [14], and drawing inappropriate or unsupported conclusions [22, 23].

**Fig. 1:**
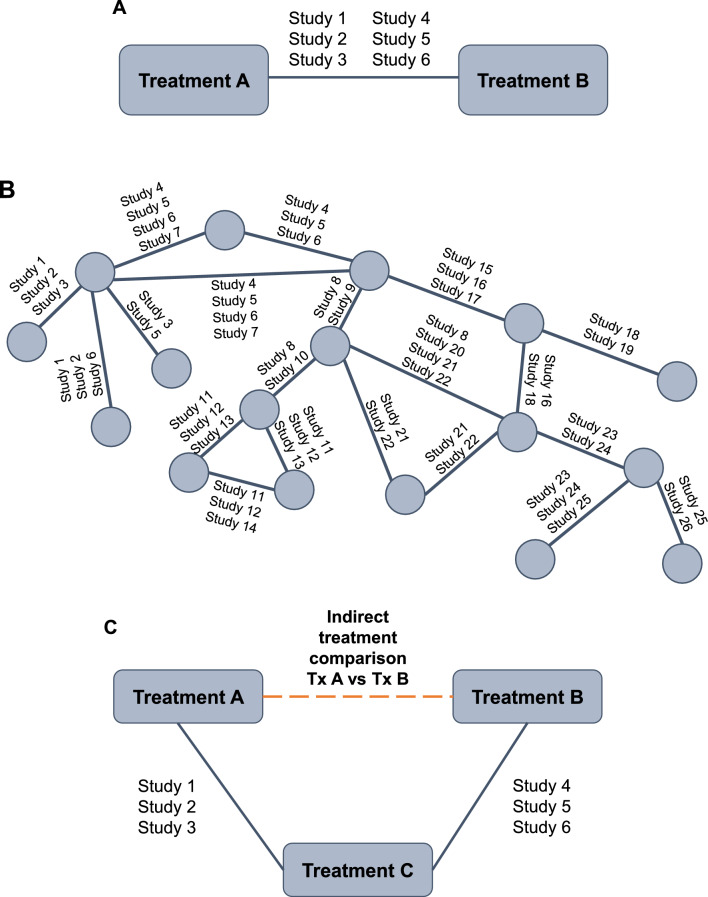
**A** Pairwise meta-analysis; **B** Network meta-analysis; **C** Indirect treatment comparison. *Tx* treatment

Meta-analysis of RCTs is now widely used to provide a summary measure of effect for an individual treatment as part of evidence synthesis [13]. Pairwise meta-analysis compares the efficacy or safety of two treatments that have been directly compared in head-to-head clinical trials, assuming the population and outcomes are comparable across trials [13, 24, 25] (Fig. 1A). A “network” diagram is constructed, which consists of “nodes” representing interventions and “lines” representing available direct comparisons between interventions (Fig. 1B) [26]. The methodology for performing pairwise meta-analysis is well established and HCPs are familiar with the outputs seen in publications such as Cochrane reviews [27]. All evidence comparing the two treatments is combined and statistical methods are used to calculate a “pooled treatment effect”, which can help inform comparative efficacy and/or safety of interventions. Common measures generated via meta-analysis include odds ratio (OR; odds of an event in the treatment group vs odds of the event in the control group); relative risk (absolute risk in the treatment group vs absolute risk in the control group); and risk difference (difference between the observed risks [proportions of individuals with the outcome of interest] in the treatment group and the control group).

In our example, a range of molecules in different therapeutic classes and different inhaler devices are available for the treatment of COPD [28] and new therapies based on combinations of these molecules have been developed and approved over recent years. Assessing the relative efficacy and effectiveness of these treatments within and between classes of monotherapy, dual, and triple therapy is challenging but important.

Sometimes, RCTs are not available to inform clinically important comparisons, such as the comparative efficacy of single-inhaler triple therapies for the treatment of COPD, and it is highly unlikely any will be undertaken. To address this problem, meta-analysis methods have been developed, which allow indirect comparisons of treatments by assessing their relative efficacy versus a common comparator using data from multiple studies [24, 29, 30] (Fig. 1C). In effect, this methodology allows researchers and decision makers to ask additional research questions beyond those originally studied. These indirect treatment comparisons (ITCs) are increasingly used by HTA bodies that are interested in the costs and benefits of the entire algorithm of treatments available (e.g., Pharmaceutical Benefits Advisory Committee in Australia, Canadian Agency for Drugs and Technologies in Health, NICE in the United Kingdom) [13, 31–33]. ITCs are able to inform decision makers of the relative effects of different medicines on individual outcomes, and provide a hierarchy of competing treatments, without compromising the rigor of the original RCT.

A pairwise meta-analysis of all relevant RCTs may be judged as being the highest level of evidence (if the analysis is of sufficient design quality) [5, 34]. Network meta-analysis (NMA) allows the simultaneous analysis of direct (head-to-head) and indirect (through a common comparator) data and is less prone to confounding bias than cohort or observational studies [24, 35].

With an increasing use of ITCs to inform clinical decisions, it is important that HCPs are able to critically appraise published analyses. Therefore, the purpose of this tutorial is to fill an important gap in the literature surrounding this topic, by providing an overview of NMA as an evidence synthesis method with a worked example of COPD pharmacotherapy. This tutorial will outline key considerations when planning, conducting, and interpreting NMAs. The tutorial will end with a simple illustrative example of different NMA methodologies using simulated data to illustrate their impact on the conclusions.

### The basics of evidence synthesis

#### Step 1: a systematic literature review

Prior to conducting a meta-analysis, all relevant RCTs in the research area must be identified through a systematic literature review (SLR). This ensures that all relevant studies are systematically identified for inclusion or exclusion in the analysis.

The SLR should follow best practice methodology, including a priori registration with the International Prospective Register of Systematic Reviews (PROSPERO) [36], and should be communicated using the Preferred Reporting Items for Systematic Reviews and Meta-Analyses (PRISMA) guidelines [37]. Cochrane, the gold standard, recommends that the search should be based on a pre-defined search string (specific for each database) and all records identified from the searches must be evaluated for their eligibility for inclusion, usually defined by the research question of interest [38]. Research questions are defined using the population, intervention, comparator, outcome, setting (PICOS) framework (Table 1). Other criteria, such as time horizon and language of studies, should also be pre-specified before the SLR is carried out.

**Table 1:** PICOS criteria

Population	Adults or children, comorbidities permitted
Intervention	Drug or drug combination of interest
Comparator	Active treatment or placebo
Outcome	e.g., change from baseline in forced expiratory volume in 1 s
Setting	Study design, duration, location/country

A non-systematic review introduces a high risk of bias, even before an ITC is completed. For example, exclusion of studies based on their design (e.g., those with placebo or no treatment arms) or restricting the inclusion of trials to those undertaken in a particular location or time period can have a significant effect on the conclusions of the analysis [39]. Theoretically, if the excluded trials are similar to those included, their omission will not have any systematic impact on the estimates, although it will lead to wider confidence intervals. However, if the excluded trials are different from those included, their omission may cause an over- or under-estimation of treatment effect. As such, the research question must be closely related to the inclusion and exclusion criteria, and all of these elements impact the application of study conclusions.

#### Step 2: data extraction and meta-analysis

Once all relevant studies have been identified, data should be extracted, and a quality assessment/risk of bias completed. A gold standard framework for assessing risk of bias is the Revised Cochrane risk-of-bias tool for randomized trials (RoB 2) [40]. Bias can occur when there are flaws in the design, conduct, analysis, and reporting of randomized trials, causing the study findings to be underestimated or overestimated. This is important for transparency of results; if a large number of included studies are deemed as having a high risk of bias, then overall findings of any combination of these studies should be interpreted with caution [41]. It is recommended that quality assessment and data extraction should be completed independently by at least two reviewers [42].

### An overview of the different approaches to network meta-analysis

NMA/multiple treatment comparison (MTC) allows the simultaneous evaluation of direct and indirect evidence across multiple treatments and studies (example in Fig. 1B). Using NMA, the identity of each treatment can be preserved (i.e., different doses and/or co-treatments), with no requirement to combine (pool) different treatment doses or combinations [43]. Other treatments that are not necessarily of interest to the research question can be included in the network of comparisons to provide additional evidence (e.g., to ‘connect’ other treatments into the network that otherwise would not be connected by providing a common comparator) [43]. All treatments in an NMA can be compared, providing they are linked (either directly or indirectly) in the final network. In some cases, the researcher may wish to compare treatments that are not linked either directly or indirectly within the study network. In this case, other methods such as matching-adjusted indirect comparisons (MAIC) can be used to make indirect comparisons [11].

### Key assumptions of NMA

Four assumptions are fundamental to NMA: similarity, transitivity, consistency, and homogeneity [4, 11, 26]. An overview of these assumptions is shown in Table 2.

**Table 2:** Key assumptions of NMA

Assumption	
Similarity	Are the studies included in the NMA similar enough in terms of research question (PICOS) to be pooled together?
Transitivity	Are there any effect modifiers (patient or study characteristics) known or thought to influence the treatment effect? If so, are there no systematic differences in the distribution of effect modifiers between the included trials?
Consistency	For mixed comparisons, is there agreement between the direct (head-to-head) and indirect (via a common comparator) evidence within the network?
Homogeneity	Are there no imbalances in population, interventions, outcomes, or study design across direct and indirect comparisons within the NMA?

*NMA* network meta-analysis; *PICOS* population, intervention, comparator, outcome, setting

#### Similarity

Studies included in an NMA must be similar. Similarity includes both clinical and methodological similarity and is based on clinical judgement and knowledge rather than statistical methods. Visualizing relevant patient characteristics (e.g., age, sex, and disease severity) across all trials included in the NMA using a summary table or showing covariate distribution via a scatter plot can help identify dissimilarity between studies (i.e., with outlying data points potentially indicating a violation of the similarity assumption) [4, 44]. Studies brought together in a network must have a similar research question (PICOS) to be pooled together without affecting effect estimations (i.e., the estimated impact of a treatment on the outcome of interest or on the association between variables). If two studies are adequately similar (e.g., mean age ranging between 45 and 60 years), the relative effect of one treatment versus placebo should remain unchanged if tested under the conditions of the other treatment versus placebo.

#### Transitivity

Transitivity implies that there are no systematic differences between the included comparisons other than the interventions being compared [26]. For example, intervention A must be similar when it appears in A versus B studies and A versus C studies with respect to all patient and study characteristics that may affect the two relative effects. Although clinical and methodological differences between studies are inevitable, prior to conducting NMA, it should be assessed whether such imbalances are considered large enough to potentially violate the transitivity assumption (e.g., the degree of lung function impairment can heavily impact results in COPD—if some studies contain mostly patients with mild lung function impairment and others include patients with more severe lung function impairment, the transitivity assumption may be violated) [26].

#### Consistency

**Fig. 2:**
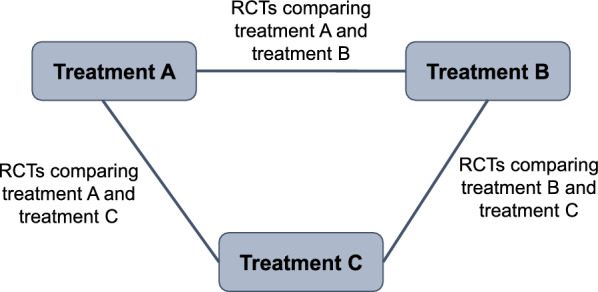
A closed loop with three treatments. *RCT* randomized controlled trial

Networks should be consistent, i.e., there should be agreement between direct (RCT) and indirect (based on a common comparator) evidence within a network [44]. Combining inconsistent evidence is inappropriate and may lead to a biased result—either an under- or overestimation of treatment effect. For closed loops within an NMA (i.e., both direct and indirect comparisons are possible; Fig. 2), inconsistency assessment can be conducted. If both direct and indirect estimations are aligned, the network can be considered consistent.

#### Homogeneity

Studies collated in an SLR will inevitably have some level of variability between them, in terms of patient population, interventions/outcomes of interest, or study design/methodological differences. Even when selected using systematic criteria, significant differences in effect modifiers can still be present. Studies may also differ in the way in which the outcomes were measured or defined, the concomitant medications allowed, the length of follow-up, or the timeframe during which the studies were conducted. As in pairwise analyses, homogeneity between studies included in an NMA must be considered. Homogeneity can be assessed in cases where identical treatment comparisons are made and multiple data sources are available. Imbalances in population, interventions, outcomes, and study design across direct and indirect comparisons in an NMA can lead to biased indirect estimations [45, 46].

### Overview of NMA methods

There are two common frameworks of NMA: frequentist (including Bucher ITC) and Bayesian. The most frequently used method in the literature is Bayesian NMA, followed by frequentist NMA, and then Bucher ITC. Bucher ITC is based on simple equations whilst frequentist and Bayesian NMA are based on more complex methods (generalized linear models).

#### Bucher ITC

The method described by Bucher and colleagues in 1997 is an ITC-based approach using simple equations (no statistical model is required) [47]. The indirect comparison of treatment A versus treatment B is estimated by comparing the treatment effects of treatment A and treatment B relative to a common comparator (treatment C; example Fig. 1C) [47]. This allows the comparison of treatments with no head-to-head evidence, whilst preserving the randomization of the original RCTs. This is often the method of choice if evidence is limited (e.g., comparison of just two interventions [48–50]). For example, in the case of three treatments (treatment A, B, and C), the indirect treatment effect of treatment A versus treatment B could be estimated as the treatment effect of A versus a common comparator (treatment C) minus the treatment effect of B versus treatment C [47]. No pooled standard error or standard deviation can be calculated (see Additional file 1, section 1.1). This method may also be the most appropriate if potential effect modifiers vary between studies, and risk introducing bias into the analysis [51]. In larger networks of evidence, indirect comparisons of interventions connected through longer paths can be conducted through multiple steps. However, adding many steps between treatments increases the uncertainty of the estimation.

The advantage of the Bucher ITC is that it is based on simple equations and relatively straightforward to conduct. A key limitation is its unsuitability for performing ITC with more complex networks of treatments with multi-arm studies. The Bucher ITC method is recommended by multiple HTA organizations as a preferred approach for conducting cross-trial ITCs [52]. See Additional file 1, section 1.1 for further information. Recent examples of studies conducted using a Bucher ITC approach in a non-COPD context include Akkoç 2023 [51], Cruz 2023 [50], Merkel 2023 [52], and Pinter 2022 [49].

#### Frequentist NMA

Frequentist NMA uses the approach most familiar to clinicians, in which measures are thought to have a fixed, unvarying (but unknown) value, without a probability distribution. Frequentist methodologies calculate confidence intervals for the value, or significance tests of hypotheses concerning it. Frequentist NMA is based on generalized linear models and uses weighted least squares regression (LSR; see Additional file 1, section 1.2 for further details).

Frequentist analysis is based solely on observed data. Hypothesis testing is conducted, with the null hypothesis being ‘no statistically significant difference between treatments’. Results are presented as estimated relative effects (mean difference, OR, etc., and a 95% confidence interval [CI; i.e., if the experiment was repeated 100 times, the true value would be covered by the interval 95 times]). P-scores can be calculated to rank treatments and results are interpreted as showing a statistically significant difference or absence thereof. Frequentist analysis is considered more conservative than a standard Bayesian NMA and corresponding 95% intervals are usually narrower.

A frequentist analysis can be implemented relatively straightforwardly using R, Stata, or Python, and there are several packages available, which make the analysis easier. The simplicity of the model can be a deciding factor in choosing frequentist over a Bayesian approach [53]. Advantages of the frequentist method are its suitability for sparse networks of evidence and the fact that the interpretation of classical statistics is more familiar to clinicians. Providing heterogeneity is moderate or low, the estimation bias is considered lower using a frequentist model than with other methods [54]. The main limitation is the inability to incorporate any additional information that may already be known about the parameter of interest (e.g., previously observed evidence from pilot or observational studies obtained through expert clinician opinion) into the analysis. Recent examples of studies conducted using a frequentist approach in a non-COPD context include: Karam [55], Lampl [53], Recchia [56], Shen [57], and Zhang [54].

#### Bayesian NMA

Bayesian methods are based on the idea that unknown quantities, such as forced expiratory volume in one second (FEV_1_) differences between treatments, have probability distributions. Bayesian NMA is also based on generalized linear models; however, the Bayesian approach is deemed more flexible than the frequentist approach as it also allows the incorporation of additional information into the model, in the form of prior distributions or ‘priors’. A prior is any external information that is already known or believed about the parameter of interest (for example, additional observational study data on the distribution of change from baseline in FEV_1_), and it represents the uncertainty about the parameter of interest before the current data are examined. The prior distribution is then updated to produce the posterior distribution by ‘learning’ from the data through an application of Bayes’ theorem [58] (see Additional file 1, section 1.3 for further details). The resulting posterior distribution is the distribution of the parameter of interest.

In contrast to the frequentist approach, during Bayesian analysis, no hypothesis testing takes place. The comparability of treatments can be shown directly but there is no ‘statistical significance’; treatments are deemed comparable, or one treatment is considered favorable/unfavorable over another. Ranking of treatments can be based on the surface under the cumulative ranking (SUCRA), a numeric presentation of the overall ranking with numbers ranging from 0 to 100%. Larger SUCRA numbers represent higher ranked interventions in the network. Results are presented as summaries from the posterior distribution, which can be the mean or median difference (or OR) and their 95% credible interval (CrI; the interval for which there is a 95% probability that the values of the treatment effect will lie within).

Bayesian analysis cannot be conducted without specification of a prior distribution, and these must be selected for basic parameters (e.g., treatment effect) and between-trial variance (in the case of a random effects model—see below section). See Additional file 1, section 1.3 for further information on selection of priors.

The ability to incorporate priors is considered an advantage of Bayesian analyses. The output can also be considered more natural in the context of decision making—i.e., it is possible to rank the orders of treatments. However, Bayesian analysis has a number of disadvantages and weaknesses; it is more computationally challenging than a frequentist approach, and a major criticism is that elicitation of priors can be difficult and subjective. In addition, data sparsity can lead to unrealistically wide CrIs.

Examples of recently published studies conducted using a Bayesian approach in a non-COPD context include: Birkinshaw [59], Chang [60], Panaccione [61], Schettini [62], and Wang [63].

### Summary of pairwise comparisons in NMA

Comparative intervention effect estimates can be presented using a square matrix known as a league table [44]. The league table shows relative effectiveness of all pairs of interventions examined along with their 95% CI or CrI. Effect estimates can be graphically represented using forest plots with 95% CIs or CrIs.

### Fixed effects versus random effects models

NMAs are usually either based on a fixed effects (FE) or random effects (RE) model (Table 3). An FE model assumes that the relative treatment effect of one treatment compared with another is the same across all trials containing those treatments (i.e., any variation in effect size between studies is due to within-study estimation error). An RE model assumes that the effect size varies between studies (i.e., the studies represent a distribution of effect sizes, and the aim of the analysis is to estimate the mean of the distribution) [64]. A ‘weight’ is assigned to each study within an NMA, which reflects the precision of the individual study estimate and therefore, the relative contribution of each study to the overall pooled result (i.e., more precise studies will contribute more to the overall estimate). In an FE model, the study weight is based solely on within-study variance. In an RE model, study weights consider between-study as well as within-study variance. This means that the relative weights of individual studies will be more alike under an RE model than they are under an FE model [64]. An RE model is more appropriate in many cases as there are differences in study and patient characteristics between the combined studies.

**Table 3:** Fixed effect model versus random effect model

	Fixed effects model	Random effects model
Treatment effect	Assumed that the treatment effect of one agent compared with another is the same across all trials containing those treatments	Assumed that the treatment effect size varies between studies
Study weights	Based upon within-study variance only	Based upon within-study and between-study variances
Use	If there are a small number of studies with large sample sizes (i.e., sparse evidence base) If there are too few studies to accurately estimate between-study variance	If substantial heterogeneity in study or patient characteristics between the combined studies is suspected

Source [64]

### Potential approaches to deal with heterogeneity or a sparse evidence base

A Chi-squared test can be used to assess whether observed differences in results across studies are due to chance alone. A low p-value (often < 0.10) indicates evidence of heterogeneity [65]. However, care must be taken when interpreting the results of the Chi-squared test, as it has low power to detect heterogeneity in analyses containing studies with small sample sizes and/or few studies. In analyses with many studies, the test has high power to detect a small amount of heterogeneity that is not necessarily clinically important. Some heterogeneity will inevitably be present in meta-analyses, and the* I*^*2*^ statistic can be used to describe the percentage of variability in effect estimates that is due to heterogeneity rather than chance [65, 66] (see Additional file 1, section 1.4). *I*^*2*^ is derived from the Chi-squared heterogeneity statistic but is independent of the number of studies and the treatment effect metric. An *I*^2^ of 0% to 40% suggests heterogeneity might not be important; 30% to 60% suggests moderate heterogeneity; 50% to 90% suggests substantial heterogeneity; and 75% to 100% suggests considerable heterogeneity [65]. If between-study heterogeneity is suspected within a network, use of an RE model should be considered [67]. If there is a large number of studies, meta-regression can also be used to investigate whether particular covariates (i.e., potential effect modifiers such as patient age) explain any of the heterogeneity of treatment effects seen between studies (see Additional file 1, section 1.5) [68]. Multi-level network meta-regression (ML-NMR) is a relatively recent extension of NMA that uses aggregate data along with individual patient data to adjust for differences in effect modifiers between studies [11]. In the case of few studies (with large sample sizes), the use of an FE model is considered more appropriate; RE models are not recommended if there are too few studies to accurately estimate between-study variance [64].

### Evaluating confidence in the results of an NMA

#### GRADE

The Grading of Recommendations, Assessment, Development, and Evaluation (GRADE) framework is recommended for use in NMA to assess the confidence in (or the quality of) the evidence for each main comparison [26, 69–71]. GRADE is used to rate evidence at an outcome level, rather than an individual study level [70, 71]. The certainty of the evidence is categorized as ‘high’, ‘moderate’, ‘low’, or ‘very low’ by outcome and the results are commonly reported using ‘summary of findings’ tables [69, 71].

GRADE assessments are determined via consideration of five domains: (1) risk of bias (i.e., are limitations in individual study designs or implementation large enough to lower confidence in the overall treatment effect); (2) consistency of effect (i.e., was there unexplained heterogeneity or variability of results across studies, which could affect the overall effect estimation); (3) indirectness (i.e., have only indirect comparisons been made or are the patients studied different from those for whom treatment recommendations would apply); (4) imprecision (i.e., do studies include few participants and/or few events); and (5) publication bias (i.e., how likely is it that selective reporting has occurred) [26, 69–71].

Although categorization is subjective, GRADE provides a transparent and reproducible framework for evidence grading; any judgements other than ‘high’ certainty should be justified using explanatory footnotes within the summary table [71].

### CINeMA

Confidence in Network Meta-Analysis (CINeMA) is another methodological framework that can be used to evaluate confidence in the results of an NMA [72, 73]. Although broadly based on the GRADE framework, the CINeMA approach has several conceptual differences [72, 73].

CINeMA assessments are determined by consideration of six domains: (1) within-study bias; (2) reporting bias; (3) indirectness; (4) imprecision; (5) heterogeneity; and (6) incoherence. Using the CINeMA framework, judgements are assigned to each domain (no concerns, some concerns, or major concerns). Judgements across the six domains can then be summarized to obtain a level of confidence for each treatment effect—these correspond to the GRADE categorizations: ‘high’, ‘moderate’, ‘low’, or ‘very low’.

The CINeMA framework can be applied to any NMA via use of the freely available web application [73].

### Appropriateness of NMA models

**Fig. 3:**
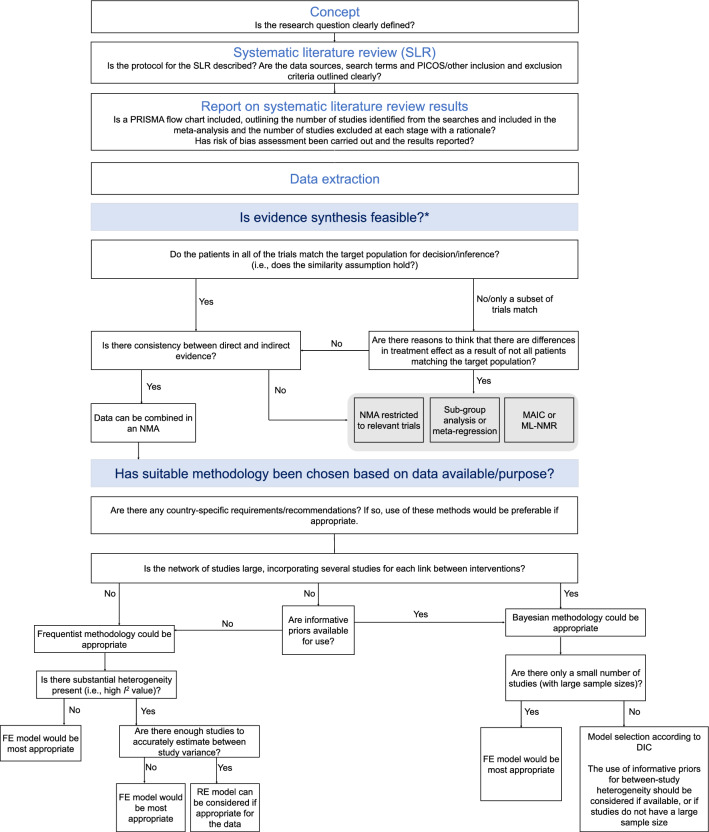
Decision framework—evidence synthesis eligibility and method selection. *Cope S, Zhang J, Saletan S, Smiechowksi B, et al. A process for assessing the feasibility of a network meta-analysis: a case study of everolimus in combination with hormonal therapy versus chemotherapy for advanced breast cancer. *BMC Medicine* 2014,12:93. *DIC* deviance information criterion, *FE* fixed effects, *MAIC* matching-adjusted indirect comparisons, *ML-NMR* multi-level network meta-regression, *NMA* network meta-analysis, *PICOS* population, intervention, comparator, outcomes, setting, *PRISMA* Preferred Reporting Items for Systematic Reviews and Meta-Analyses, *RE* random effects, *SLR* systematic literature review

A comparison of frequentist and Bayesian methodologies is shown in Table 4 and a summary of key inputs and outputs for each method is shown in Table 5. Table 6 summarizes the key steps involved in NMA and Fig. 3 outlines a framework for assessing the robustness of a study and the suitability of methods chosen, given the data.

**Table 4:** Comparison of frequentist and Bayesian approach

	Frequentist approach	Bayesian approach
NMA data input preparation	Input required for all pairwise comparisons per study (*k* (*k* − 1)/2), with *k* representing number of arms, equating to 1 for a 2-arm study, 3 for a 3-arm study, 6 for a 4-arm study etc	Input required for all comparisons to the baseline treatment, corresponding to number of arms − 1 (*k* − 1): 1 for a 2-arm study, 2 for a 3-arm study, 3 for a 4-arm study etc
Prior specification	No priors used – based solely on observed data	Prior distribution must be specified
Analysis	Straightforward implementation using available R package netmeta Statistical hypothesis testing is conducted. Output is presented as the estimates of effects and corresponding 95% CIs and associated p-values for the tests of significance	More computationally intensive, yet OpenBUGS code readily available at NICE Decision Support Unit No hypothesis testing takes place. Output is presented as the estimates of effect and corresponding 95% CrI, Bayes factor, and posterior probabilities of effect
Interpretation	Results are presented as estimated relative effects (mean difference or odds ratio) and 95% CIs, statistical significance can be determined Ranking of treatments through p-score, the frequentist equivalent to the SUCRA	Results are presented as summaries of the posterior distribution (of the mean difference or odds ratio) and 95% CrIs, no statistical significance can be determined. Results are interpreted as one treatment to be favorable/unfavorable over another treatment, or two treatments to be comparable Ranking of treatments through SUCRA. Probability of one treatment to be better than another treatment can additionally be estimated (not feasible in a frequentist approach)

*CI* confidence interval, *CrI* credible interval, *NICE* National Institute for Health and Care Excellence, *NMA* network meta-analysis, *SUCRA* surface under the cumulative ranking curve

**Table 5:** Summary of key inputs and outputs

	Frequentist approach	Bayesian approach
Is the network of evidence sparse? (<5 studies)	Works well	Does not work well for standard non-informative priors but works well for informative priors Non-informative priors could result in unrealistically wide credible intervals
Is prior specification justified?	No priors used—based solely on observed data	Non-informative if enough data are available, informative in the case of sparse data, choice of suitable distributions and additional information such as expert clinician opinion
Are there few large studies of high quality?	Consider FE model	Consider FE model
Are there country-specific regulations?	Required by German G-BA and Australian PBAC	Preferred by NICE in the UK
Interpretation	Statistical significance or the absence thereof	One treatment favorable/unfavorable over another treatment, or two treatments comparable Often falsely interpreted as significant or not significant; common misconception

*FE* fixed effects, *G-BA* Federal Joint Committee, *NICE* National Institute for Health and Care Excellence, *PBAC* Pharmaceutical Benefits Advisory Committee

**Table 6:** Summary of steps involved in NMA

Step	Further information/considerations	Additional resources
Systematic literature review	Prospective registration with PROSPERO Well-defined research question using the PICOS framework Searches carried out using a pre-defined search string (specific to each database) Systematic inclusion/exclusion of studies per the research question	PROSPERO: [36] Cochrane handbook: [38]
Data extraction and network generation	Quality/risk of bias assessment Treatment network defined	RoB 2 tool: [40] Cochrane handbook: [26]
Assessment of NMA assumptions	*Similarity*: similarity in PICOS criteria of all included studies *Transitivity*: no systematic differences in the distribution of effect modifiers between included studies *Consistency*: agreement between direct and indirect evidence within the network *Homogeneity*: no imbalances in PICOS across direct and indirect comparisons within the network	Cochrane handbook: [26]
Conducting an NMA	Appropriate statistical model used for the available data and/or any specific country requirements Justified use of FE vs RE methods Appropriate presentation of results For frequentist analysis: estimates of effects and corresponding 95% CIs and associated p-values For Bayesian analysis: estimates of effects and corresponding 95% CrIs	Cochrane handbook: [26] Bucher 1997: [47] Netmeta: [74] NICE DSU: [12]
Interpretation of NMA findings	Appropriate and careful interpretation of findings For frequentist analysis: ranking of treatments through p-scores. Can be interpreted as statistical significance or absence thereof For Bayesian analysis: ranking of treatments through SUCRA. No significance testing Use of the GRADE framework to assess the confidence in the evidence	Cochrane handbook: [26] NMA worked example for clinicians: [75] GRADE resources: [69, 70]
Reporting of NMA findings	Communicated following the PRISMA guidelines for NMA	PRISMA: [37]

*CI* confidence interval, *CrI* credible interval, *FE* fixed effects, *GRADE* Grading of Recommendations, Assessment, Development and Evaluation, *NICE* National Institute for Health and Care Excellence, *NMA* network meta-analysis, *PICOS* population, intervention, comparator, outcome(s) and setting, *PRISMA* Preferred Reporting Items for Systematic Reviews and Meta-Analyses, *PROSPERO* International Prospective Register of Systematic Reviews, *RE* random effects, *RoB* risk of bias, *SUCRA* surface under the cumulative ranking curve

### Illustrative example of different NMA methods in COPD

**Fig. 4:**
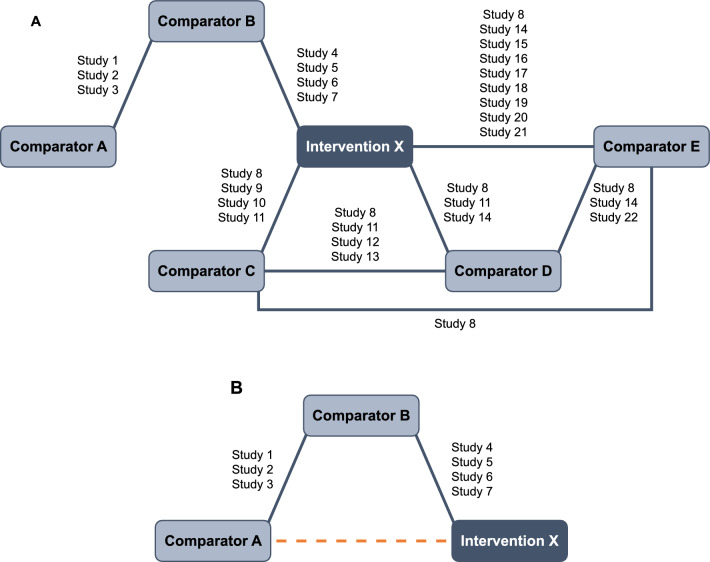
Illustrative example: **A** Network of evidence and **B** Bucher ITC network. *ITC* indirect treatment comparison

We have used a simulated data set to show the results from the three different statistical frameworks (frequentist NMA, Bayesian NMA, and Bucher ITC). The example compares the efficacy of fictitious “intervention X” with five comparators (interventions A–E) on change in FEV_1_ from baseline. The setting was defined as a large evidence base (i.e., more than three interventions in total and more than one study informing most links in the network; Fig. 4A). Data were simulated using a normal random number generator in R, with the same mean for pairwise comparisons on the same interventions, and standard deviations (SDs) ranging from 7 to 20 to incorporate a realistic amount of heterogeneity in the data. The mean was estimated from real FEV_1_ data, and then varied for the different pairwise comparisons, ranging from 40 to 65. The amount of heterogeneity in the simulated data was set at a realistic, moderate level (*I*^2^ = 55%). Further details regarding data simulation and the final data set are shown in Additional file 1 (section 1.6 and Tables S1–S3).

**Fig. 5:**
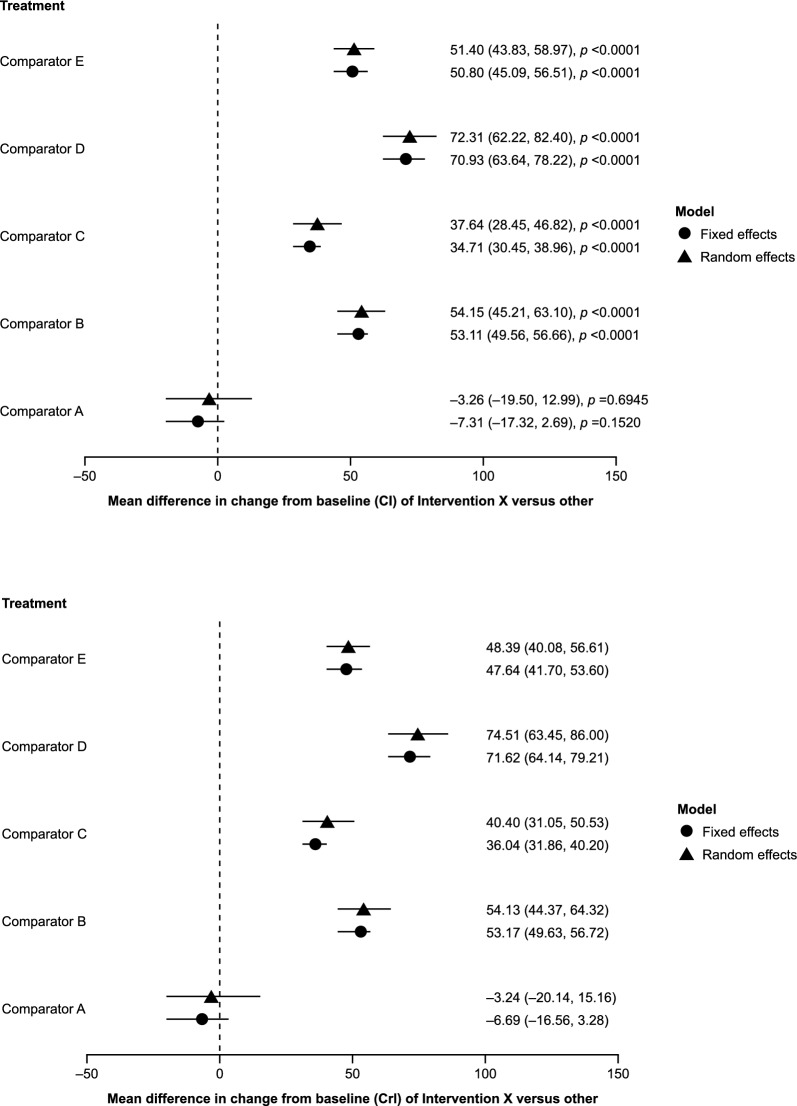
Illustrative example: Mean change from baseline FEV_1_: **A** frequentist and **B** Bayesian comparison. *Abbreviations*: *CI* confidence interval, *CrI* credible interval, *FEV*_*1*_ forced expiratory volume in 1 s

**Fig. 6:**
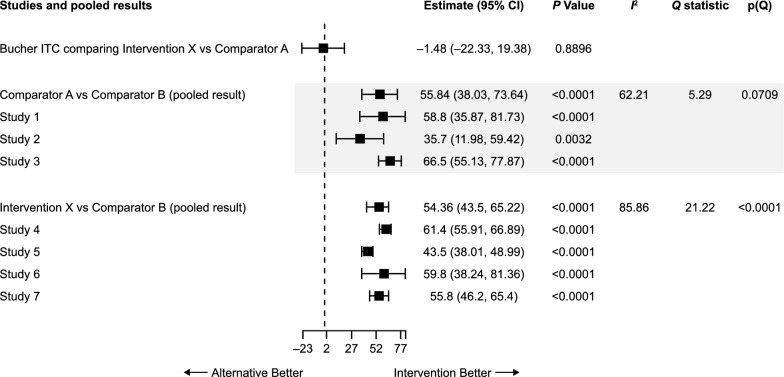
Illustrative example: Bucher ITC—intervention X versus Comparator A. *CI* confidence interval, *ITC* indirect treatment comparison

Results of the frequentist and Bayesian analyses are shown in Fig. 5. The FE and RE models were compared to account for between-study heterogeneity. Although some differences in point estimates were seen, the overall results of the analyses were similar using both frequentist and Bayesian frameworks as well as both FE and RE models. The ranking of interventions using each method is shown in Table 7. Despite minor numerical differences, the overall ranking of treatments was consistent across all analyses. Pairwise results from each analysis and the “probability of intervention X being better than the comparator” for the Bayesian analyses are shown in Additional file 1 (Tables S4 and S5, respectively). The section of the network used for the Bucher ITC is shown in Fig. 4B. The result of the indirect comparison of intervention X versus comparator A is shown in Fig. 6 and Additional file 1, Table S6. Results were consistent with frequentist and Bayesian approaches.

**Table 7:** Illustrative example: ranking of treatments

Frequentist FE model		Frequentist RE model	
Intervention	*p*-score	Intervention	*p*-score
Comparator A	0.984798	Comparator A	0.930552
Intervention X	0.815202	Intervention X	0.869446
Comparator C	0.600000	Comparator C	0.597584
Comparator E	0.349816	Comparator E	0.336729
Comparator B	0.250183	Comparator B	0.264845
Comparator D	1.71E − 06	Comparator D	0.000843

Bayesian FE model		Bayesian RE model	
Intervention	SUCRA	Intervention	SUCRA
Comparator A	0.981027	Comparator A	0.92899
Intervention X	0.818973	Intervention X	0.870971
Comparator C	0.599903	Comparator C	0.577122
Comparator E	0.388257	Comparator E	0.382042
Comparator B	0.211838	Comparator B	0.23979
Comparator D	1.9E − 06	Comparator D	0.001084

*FE* fixed effects, *RE* random effects, *SUCRA* surface under the cumulative ranking curve

In summary, the illustrative example demonstrates that Bucher ITC, frequentist NMA, and Bayesian NMA, using both FE and RE models, give results that are similar and are in alignment. Although there were some numerical differences in point estimates, and the width of the intervals differed slightly across analyses, all conclusions drawn were identical. If the evidence base is large, and non-informative priors are used in the Bayesian model, the results obtained using frequentist and Bayesian methods are comparable. This example should be considered within the limitation of using simulated data; for example, it is not possible to present a GRADE summary of findings for this analysis.

## Conclusions

A range of molecules in different therapeutic classes are available for the treatment of COPD; assessing the relative effectiveness of these molecules within and between classes can be challenging. There are various ways of synthesizing the available efficacy data of different interventions when head-to-head studies do not exist. Frequentist (including Bucher ITC) and Bayesian are two commonly used NMA frameworks. Network sparsity, authority requirements, and general preference may influence the choice of statistical model, and authors should be able to justify the method selected. HCPs can assess the appropriateness of the model and the assumptions that underpin it using the information in this tutorial. However, providing the methods are applied correctly, the outcome should be consistent regardless of which method is chosen.

## Supplementary Information

Additional file 1: Fig. S1. Supplementary appendix, sections 1.1–1.6. [NMA methods – additional information and details of illustrative example data simulation.] Table S1. [Illustrative example: frequentist data.] Table S2. [Illustrative example: Bayesian data.] Table S3. [Illustrative example: Bucher ITC data.] Table S4. [Illustrative example: pairwise results.] Table S5. [Illustrative example: probability of being better than comparator – Bayesian analysis.] Table S6. [Illustrative example: indirect comparison of intervention X versus comparator A] Figure S1. [LSR model.]

## Data Availability

No datasets were generated or analyzed during the current study.

## Abbreviations

CI: Confidence interval
CINeMA: Confidence in Network Meta-Analysis
COPD: Chronic obstructive pulmonary disease
CrI: Credible interval
DIC: Deviance information criterion
FE: Fixed effects
FEV_1_: Forced expiratory volume in 1 s
GRADE: Grading of Recommendations, Assessment, Development, and Evaluation
HCP: Healthcare professional
HTA: Health technology assessment
ITC: Indirect treatment comparison
LSR: Least squares regression
MAIC: Matching-adjusted indirect comparisons
ML-NMR: Multi-level network meta-regression
MTC: Multiple treatment comparison
NICE: National Institute for Health and Care Excellence
NMA: Network meta-analysis
OR: Odds ratio
PBAC: Australian Pharmaceutical Benefits Advisory Committee
PICOS: Population, intervention(s), comparator(s), outcome(s), setting
PRISMA: Preferred Reporting Items for Systematic Reviews and Meta-Analyses
PROSPERO: International Prospective Register of Systematic Reviews
RCT: Randomized controlled trial
RE: Random effects
SD: Standard deviation
SLR: Systematic literature review
SUCRA: Surface under the cumulative ranking
TSD: Technical Support Document
Tx: Treatment
