# Supplementary material for: Assessing the comparative effects of interventions in COPD: a tutorial on network meta-analysis for clinicians
Source: Respir Res. 2024 Dec 21;25:438. doi: 10.1186/s12931-024-03056-x (PMC11663313; doi:10.1186/s12931-024-03056-x)
Supplement: Supplementary file 1 — Additional file 1: Fig. S1. Supplementary appendix, sections 1.1–1.6. [NMA methods – additional information and details of illustrative example data simulation.] Table S1. [Illustrative example: frequentist data.] Table S2. [Illustrative example: Bayesian data.] Table S3. [Illustrative example: Bucher ITC data.] Table S4. [Illustrative example: pairwise results.] Table S5. [Illustrative example: probability of being better than comparator – Bayesian analysis.] Table S6. [Illustrative example: indirect comparison of intervention X versus comparator A] Figure S1. [LSR model.] [file 12931_2024_3056_MOESM1_ESM.docx]

**Supplementary Appendix**

**1.1 Overview of NMA Methods: Bucher ITC – additional information**

*Bucher ITC – additional methods*

In Bucher ITC, no pooled standard error can be calculated; hence the variance is the sum of the direct variances. For example, using the example in Fig. 1C, the variance of treatment effect AB = variance of treatment effect AC + variance of treatment effect BC. Standard error of the treatment effect AB = √(Standard error of the treatment effect AC)^2^ + (Standard error of the treatment effect BC)^2^.

*Bucher ITC – HTA recommendations*

The Australian Pharmaceutical Benefits Advisory Committee (PBAC) mention the Bucher ITC method as being the most widely accepted method, and recommend that submissions can include other NMA methods as supplementary evidence [1]. German Institute for Quality and Efficiency in Health Care (IQWiG) cite Bucher’s method in particular to be most appropriate for indirect comparisons in health economics [2]. Other methodologies, such as NMA, are also accepted by the German health authorities in certain circumstances, for example, if a detailed justification is given [2]. However, the Bucher ITC method is usually preferred.

**1.2 Overview of NMA Methods: frequentist NMA – additional information**

Frequentist NMA – additional methods

Frequentist NMA is based on weighted least squares regression (LSR). An LSR finds the best model by minimizing the total distance between the model prediction of the mean response value (after inputting values of the predictors and the categorical variables), and the observed outcome data (Supplementary Fig. S1). In weighted LSR, the uncertainty about the data points is incorporated into the regression – a study with a larger variance will contribute less to the overall estimate than a study with a smaller variance. The residuals of a study are weighted by the study weight, which is the inverse of the corresponding within-study variance in a FE model, or the sum of within-study variance and between-study variance in a RE model. This approach is based on the notion of repeated experiments (i.e., if the experiment, such as a fair coin toss, were to be replicated many times, we could estimate the unknown probability of a head versus a tail) and assumes that the parameter of interest takes a single fixed value (e.g., measuring FEV_1_ in a patient 1,000 times and then estimating the mean value and 95% confidence interval [CI] from the 1,000 values).

**1.3 Overview of NMA Methods: Bayesian NMA – additional information**

*Bayesian NMA – additional methods*

In Bayesian NMA, the prior distribution is updated into the posterior distribution by learning from the data. If the prior and the data are conflicting, this may result in more uncertainty about the parameter. Markov Chain Monte Carlo (MCMC) simulation is used with a large enough number of simulations to ensure convergence (usually 10,000–100,000 simulations depending upon the complexity of the NMA model). With each simulation, the prior ‘learns’ more from the data and knowledge is increased. Relative goodness-of-fit of models can be assessed using deviance information criterion (DIC). DIC estimates the effective number of parameters by the difference of the posterior mean of the deviance and the deviance at the posterior mean. This coincides with the number of independent parameters in FE models with flat priors. Absolute model fit to the data could for example be assessed via posterior predictive checks. Assessing model fit according to DIC can also help identifying the most suitable priors for the data available.

*Bayesian NMA – prior selection*

Often, ‘non-informative’ priors on between-study heterogeneity parameters are selected. These are usually based on uniform distributions (in which all outcomes are assumed to be equally likely) or inverse gamma distributions (which are right-skewed). [3-6]. A right-skewed distribution has a peak to the left and a long tail to the right, and the median is lower than the mean. Such distribution is especially suitable for parameters with mainly low values and just a few high values, such as income. Non-informative distributions imply that very little information is known on the extent of the between-study heterogeneity present in the analysis. Non-informative priors on treatment effects are usually based on normal distributions. A non-informative prior will have minimal impact on the posterior distribution and in such cases the results of Bayesian and frequentist analyses would be expected to be very similar. Non-informative priors can be used in cases where enough data are available to update the prior distribution into a posterior distribution. If the evidence is too sparse, the method cannot update the priors properly, resulting in unrealistically wide 95% CrIs, preventing a treatment being shown as favorable over a comparator. From experience, having around 10 studies in the NMA with at least 3 studies per link in the network is sufficient to prevent an unrealistically high amount of uncertainty in the results when using NICE Decision Support Unit models. Alternatively, an ‘informative prior’ can be used if available. This is a prior distribution based on previously known or believed information, which will draw the parameter towards this information. Characterized by smaller variances, informative priors express specific information about a variable; the more specific the prior is, the more influential it will be.

Prior elicitation is crucial when the data are poor. Priors can be derived in several ways: 1) they could be previously observed distributions of treatment effect; 2) they can be empirically derived predictive distributions on between-study heterogeneity obtained from the literature – for example, Rhodes et al. analyzed >6,000 continuous-outcome meta-analyses within the Cochrane Database of Systematic Reviews to investigate the influence of meta-analysis settings on heterogeneity by modelling study data from all meta-analyses on the standardized mean difference scale [7]; 3) they can be derived through testing of alternate distributions such as half-normal, gamma, or exponential priors and evaluating the agreement of posterior summaries based on different priors [5, 8]; 4) they can be elicited through expert clinical opinion (e.g., by interviewing a panel of specialists and synthesizing the findings); 5) they could be obtained from non-RCT studies (e.g., observational or pilot studies that are not directly included in the analysis).

*Bayesian NMA – HTA recommendations*

The European Network for Health Technology Assessment (EUnetHTA) and the Member State Coordination Group on Health technology Assessment (HTA CG) acknowledge the utility of Bucher ITC, frequentist, and Bayesian methods for HTA submissions, providing that the appropriateness of the chosen model is validated [9, 10], whilst (as discussed above) guidance from The NICE Decision Support Unit suggests using a Bayesian methodology [11], although any method fulfilling the required properties is valid.

**1.4 Heterogeneity: additional information**

$I^{2 =}\binom{Q-df}{Q}$ x 100

Q is the chi squared statistic and df is its degrees of freedom

*I*^2^ describes the percentage of the variability in effect estimates that is due to heterogeneity rather than sampling error (chance)

**1.5 Potential approaches to deal with heterogeneity or a sparse evidence base: meta-regression**

Meta-regression relates the size of a treatment effect to the characteristics of included studies. This allows patient and study characteristics to be adjusted for by including study level covariates in the linear predictor of the model (assuming that the characteristic is linearly related to the outcome), thus allowing quantification of the relationship between baseline characteristics and outcomes [12]. Centering the covariate values involves calculating the difference between an overall mean from all trials and the mean from each arm of each trial, to provide the covariate value for each arm in the analysis. The meta-regression–adjusted models can be compared with the unadjusted models for each endpoint assessed, in order to understand the effect of adjusting for baseline differences between trials on the estimated treatment effects. The effect of the baseline value on the endpoint can be quantified through the covariate value produced from the analysis, which represents the impact of the covariates [12].

**1.6 Illustrative example of different NMA methods in COPD: data simulation**

Various pairwise comparisons in the model were not independent since direct and indirect comparisons can only vary to a certain extent as in real study data. Therefore, a number of pairwise comparisons were estimated through indirect comparison and not directly through random number generation; for example, for the comparison of intervention X and comparator D, XD, was estimated as the difference of XC and DC, following Bucher indirect treatment comparison (ITC). A small amount of heterogeneity through a Normal random number generator with small SD of 5 was then added. This approach has been followed throughout to ensure that there were no unrealistically high discrepancies between direct and indirect comparisons.

**Supplementary Table S1** Illustrative example: frequentist data

|  | **Treatment effect** | **Standard error of treatment effect** | **Treatment 1** | **Treatment 2** | **Study** |
| --- | --- | --- | --- | --- | --- |
| 1 | 58.8 | 11.7 | Comparator A | Comparator B | Study 1 |
| 2 | 35.7 | 12.1 | Comparator A | Comparator B | Study 2 |
| 3 | 66.5 | 5.8 | Comparator A | Comparator B | Study 3 |
| 4 | 61.4 | 2.8 | Intervention X | Comparator B | Study 4 |
| 5 | 43.5 | 2.8 | Intervention X | Comparator B | Study 5 |
| 6 | 59.8 | 11 | Intervention X | Comparator B | Study 6 |
| 7 | 55.8 | 4.9 | Intervention X | Comparator B | Study 7 |
| 8 | 32.9 | 2.5 | Intervention X | Comparator C | Study 9 |
| 9 | 66.1 | 8.7 | Intervention X | Comparator C | Study 10 |
| 10 | 47.3 | 11.6 | Intervention X | Comparator E | Study 15 |
| 11 | 58.9 | 10.7 | Intervention X | Comparator E | Study 16 |
| 12 | 50.2 | 9.2 | Intervention X | Comparator E | Study 17 |
| 13 | 50.6 | 11.6 | Intervention X | Comparator E | Study 18 |
| 14 | 56.6 | 7.3 | Intervention X | Comparator E | Study 19 |
| 15 | 37.9 | 7.7 | Intervention X | Comparator E | Study 20 |
| 16 | 52.6 | 9.4 | Intervention X | Comparator E | Study 21 |
| 17 | 44.7 | 9.6 | Comparator C | Comparator D | Study 12 |
| 18 | 36.5 | 14.9 | Comparator C | Comparator D | Study 13 |
| 19 | −22.1 | 8.7 | Comparator D | Comparator E | Study 22 |
| 20 | 33.3 | 8.9 | Intervention X | Comparator C | Study 11 |
| 21 | 68 | 9.4 | Intervention X | Comparator D | Study 11 |
| 22 | 34.7 | 8.5 | Comparator C | Comparator D | Study 11 |
| 23 | 70.6 | 8.8 | Intervention X | Comparator D | Study 14 |
| 24 | 47.8 | 9.6 | Intervention X | Comparator E | Study 14 |
| 25 | −22.8 | 9 | Comparator D | Comparator E | Study 14 |
| 26 | 33.4 | 8.9 | Intervention X | Comparator C | Study 8 |
| 27 | 66.3 | 8.5 | Intervention X | Comparator D | Study 8 |
| 28 | 56.2 | 9.4 | Intervention X | Comparator E | Study 8 |
| 29 | 32.9 | 8.7 | Comparator C | Comparator D | Study 8 |
| 30 | −10.1 | 9.5 | Comparator D | Comparator E | Study 8 |
| 31 | 22.8 | 8.8 | Comparator C | Comparator E | Study 8 |

**Supplementary Table S2** Illustrative example: Bayesian data

| **t1** | **t2** | **t3** | **t4** | **y1** | **y2** | **y3** | **y4** | **SE1** | **SE2** | **SE3** | **SE4** | ***n*_a_** | ***V*** | **Study** |
| --- | --- | --- | --- | --- | --- | --- | --- | --- | --- | --- | --- | --- | --- | --- |
| 1 | 2 | NA | NA | NA | −58.8 | NA | NA | NA | 11.7 | NA | NA | 2 | NA | Study 1 |
| 1 | 2 | NA | NA | NA | −35.7 | NA | NA | NA | 12.1 | NA | NA | 2 | NA | Study 2 |
| 1 | 2 | NA | NA | NA | −66.5 | NA | NA | NA | 5.8 | NA | NA | 2 | NA | Study 3 |
| 2 | 6 | NA | NA | NA | 61.4 | NA | NA | NA | 2.8 | NA | NA | 2 | NA | Study 4 |
| 2 | 6 | NA | NA | NA | 43.5 | NA | NA | NA | 2.8 | NA | NA | 2 | NA | Study 5 |
| 2 | 6 | NA | NA | NA | 59.8 | NA | NA | NA | 11 | NA | NA | 2 | NA | Study 6 |
| 2 | 6 | NA | NA | NA | 55.8 | NA | NA | NA | 4.9 | NA | NA | 2 | NA | Study 7 |
| 3 | 6 | NA | NA | NA | 32.9 | NA | NA | NA | 2.5 | NA | NA | 2 | NA | Study 9 |
| 3 | 6 | NA | NA | NA | 66.1 | NA | NA | NA | 8.7 | NA | NA | 2 | NA | Study 10 |
| 5 | 6 | NA | NA | NA | 47.3 | NA | NA | NA | 11.6 | NA | NA | 2 | NA | Study 15 |
| 5 | 6 | NA | NA | NA | 58.9 | NA | NA | NA | 10.7 | NA | NA | 2 | NA | Study 16 |
| 5 | 6 | NA | NA | NA | 50.2 | NA | NA | NA | 9.2 | NA | NA | 2 | NA | Study 17 |
| 5 | 6 | NA | NA | NA | 50.6 | NA | NA | NA | 11.6 | NA | NA | 2 | NA | Study 18 |
| 5 | 6 | NA | NA | NA | 56.6 | NA | NA | NA | 7.3 | NA | NA | 2 | NA | Study 19 |
| 5 | 6 | NA | NA | NA | 37.9 | NA | NA | NA | 7.7 | NA | NA | 2 | NA | Study 20 |
| 5 | 6 | NA | NA | NA | 52.6 | NA | NA | NA | 9.4 | NA | NA | 2 | NA | Study 21 |
| 3 | 4 | NA | NA | NA | −44.7 | NA | NA | NA | 9.6 | NA | NA | 2 | NA | Study 12 |
| 3 | 4 | NA | NA | NA | −36.5 | NA | NA | NA | 14.9 | NA | NA | 2 | NA | Study 13 |
| 4 | 5 | NA | NA | NA | 22.1 | NA | NA | NA | 8.7 | NA | NA | 2 | NA | Study 22 |
| 3 | 4 | 6 | NA | NA | −34.7 | 33.3 | NA | NA | 8.5 | 8.9 | NA | 3 | 4.35 | Study 11 |
| 4 | 5 | 6 | NA | NA | 22.8 | 70.6 | NA | NA | 9 | 8.8 | NA | 3 | 4.45 | Study 14 |
| 3 | 4 | 5 | 6 | NA | −32.9 | 10.1 | 33.4 | NA | 8.7 | 9.5 | 8.9 | 4 | 2.0 | Study 8 |

**Abbreviations:** NA, not applicable; *n*_a_, number of arms; SE, standard error; t, treatment; y, treatment effect; *V*, the variance of the baseline treatment in that trial (needed to adjust for the correlation in multi-arm trials – note that this variable only need to have values assigned when there are multi-arm trials)

**Supplementary Table S3** Illustrative example: Bucher ITC data

| **Study** | **Treatment effect** | **Standard error of treatment effect** | **Intervention 1** | **Intervention 2** |
| --- | --- | --- | --- | --- |
| 1 | 58.8 | 11.7 | Comparator A | Comparator B |
| 2 | 35.7 | 12.1 | Comparator A | Comparator B |
| 3 | 66.5 | 5.8 | Comparator A | Comparator B |
| 4 | 61.4 | 2.8 | Intervention X | Comparator B |
| 5 | 43.5 | 2.8 | Intervention X | Comparator B |
| 6 | 59.8 | 11 | Intervention X | Comparator B |
| 7 | 55.8 | 4.9 | Intervention X | Comparator B |

**Supplementary Table S4** Illustrative example: pairwise results

|  | **Comparator  A** | **Comparator  B** | **Comparator  C** | **Comparator  D** | **Comparator  E** | **Intervention X** |
| --- | --- | --- | --- | --- | --- | --- |
| **Frequentist fixed effects** | | | | | | |
| **Comparator A** | 0  (0, 0) | 60.42  (51.06, 69.78) | 42.02  (31.15, 52.9) | 78.24  (65.86, 90.63) | 58.12  (46.59, 69.64) | 7.31  (−2.69, 17.32) |
| **Comparator B** | −60.42  (−69.78, −51.06) | 0  (0, 0) | −18.4  (−23.94, −12.86) | 17.82  (9.72, 25.93) | −2.3  (−9.03, 4.42) | −53.11  (−56.66, −49.56) |
| **Comparator C** | −42.02  (−52.9, −31.15) | 18.4  (12.86, 23.94) | 0  (0, 0) | 36.22  (28.78, 43.66) | 16.09  (9.36, 22.83) | −34.71  (−38.96, −30.45) |
| **Comparator D** | −78.24  (−90.63, −65.86) | −17.82  (−25.93, −9.72) | −36.22  (−43.66, −28.78) | 0  (0, 0) | −20.13  (−28.07, −12.18) | −70.93  (−78.22, −63.64) |
| **Comparator E** | −58.12  (−69.64, −46.59) | 2.3  (−4.42, 9.03) | −16.09  (−22.83, −9.36) | 20.13  (12.18, 28.07) | 0 (0, 0) | −50.8  (−56.51, −45.09) |
| **Intervention X** | −7.31  (−17.32, 2.69) | 53.11  (49.56, 56.66) | 34.71  (30.45, 38.96) | 70.93  (63.64, 78.22) | 50.8  (45.09, 56.51) | 0  (0, 0) |
| **Frequentist random effects** | | | | | | |
| **Comparator A** | 0  (0, 0) | 57.41  (43.85, 70.97) | 40.89  (22.23, 59.56) | 75.57  (56.44, 94.69) | 54.66  (36.73, 72.58) | 3.26  (−12.99, 19.5) |
| **Comparator B** | −57.41  (−70.97, −43.85) | 0  (0, 0) | −16.52  (−29.34, −3.69) | 18.16  (4.67, 31.64) | −2.75  (−14.47, 8.96) | −54.15  (−63.1, −45.21) |
| **Comparator C** | −40.89  (−59.56, −22.23) | 16.52  (3.69, 29.34) | 0  (0, 0) | 34.67  (24.15, 45.19) | 13.76  (2.95, 24.57) | −37.64  (−46.82, −28.45) |
| **Comparator D** | −75.57 (−94.69, −56.44) | −18.16  (−31.64, −4.67) | −34.67  (−45.19, −24.15) | 0  (0, 0) | −20.91  (−31.54, −10.28) | −72.31  (−82.4, −62.22) |
| **Comparator E** | −54.66  (−72.58, −36.73) | 2.75  (−8.96, 14.47) | −13.76  (−24.57, −2.95) | 20.91  (10.28, 31.54) | 0  (0, 0) | −51.4  (−58.97, −43.83) |
| **Intervention X** | −3.26  (−19.5, 12.99) | 54.15  (45.21, 63.1) | 37.64  (28.45, 46.82) | 72.31  (62.22, 82.4) | 51.4  (43.83, 58.97) | 0  (0, 0) |
| **Bayesian fixed effects** | | | | | | |
| **Comparator A** | 0  (0, 0) | 59.8  (50.5, 69.1) | 42.7  (31.9, 53.4) | 78.3  (65.8, 90.8) | 54.3  (42.8, 65.8) | 6.7  (−3.3, 16.6) |
| **Comparator B** | −59.8  (−69.1, −50.5) | 0  (0, 0) | −17.1  (−22.7, −11.7) | 18.4  (10.2, 26.9) | −5.5  (−12.4, 1.4) | −53.2  (−56.7, −49.6) |
| **Comparator C** | −42.7  (−53.4, −31.9) | 17.1  (11.7, 22.7) | 0  (0, 0) | 35.6  (28.2, 43.0) | 11.6  (4.9, 18.4) | −36.0  (−40.2, −31.9) |
| **Comparator D** | −78.3  (−90.8, −65.8) | −18.4  (−26.9, −10.2) | −35.6  (−43.0, −28.2) | 0  (0, 0) | −24.0  (−32.2, −15.9) | −71.6  (−79.2, −64.1) |
| **Comparator E** | −54.3  (−65.8, −42.8) | 5.5  (−1.4, 12.4) | −11.6  (−18.4, −4.9) | 24.0  (15.9, 32.2) | 0  (0, 0) | −47.6  (−53.6, −41.7) |
| **Intervention X** | −6.7  (−16.6, 3.3) | 53.2  (49.6, 56.7) | 36.0  (31.9, 40.2) | 71.6  (64.1, 79.2) | 47.6  (41.7, 53.6) | 0  (0, 0) |
| **Bayesian random effects** | | | | | | |
| **Comparator A** | 0  (0, 0) | 57.35  (42.49, 71.41) | 43.62  (23.29, 63.31) | 77.73  (56.40, 98.22) | 51.60  (31.43, 70.42) | 3.24  (−15.16, 20.14) |
| **Comparator B** | −57.35  (−71.41, −42.49) | 0  (0, 0) | −13.75  (−27.37, 0.36) | 20.35  (5.40, 35.54) | −5.77  (−18.89, 7.02) | −54.13  (−64.32, −44.37) |
| **Comparator C** | −43.62  (−63.31, −23.29) | 13.75  (−0.36, 27.37) | 0  (0, 0) | 34.09  (22.86, 45.13) | 7.97  (−3.93, 19.14) | −40.40  (−50.53, −31.05) |
| **Comparator D** | −77.73  (−98.22, −56.40) | −20.35  (−35.54, −5.40) | −34.09  (−45.13, −22.86) | 0  (0, 0) | −26.14  (−38.08, −14.60) | −74.51  (−86.00, −63.45) |
| **Comparator E** | −51.60  (−70.42, −31.43) | 5.77  (−7.02, 18.89) | −7.97  (−19.14, 3.93) | 26.14  (14.60, 38.08) | 0  (0, 0) | −48.39  (−56.61, −40.08) |
| **Intervention X** | −3.24  (−20.14, 15.16) | 54.13  (44.37, 64.32) | 40.40  (31.05, 50.53) | 74.51  (63.45, 86.00) | 48.39  (40.08, 56.61) | 0  (0, 0) |

**Supplementary Table S5** Illustrative example: probability of being better than comparator – Bayesian analysis

|  | **Comparator A** | **Comparator B** | **Comparator C** | **Comparator D** | **Comparator E** | **Intervention X** |
| --- | --- | --- | --- | --- | --- | --- |
| **Fixed effects** | | | | | | |
| Comparator A | NA | 1 | 1 | 1 | 1 | 0.91 |
| Comparator B | 0 | NA | 0 | 1 | 0.06 | 0 |
| Comparator C | 0 | 1 | NA | 1 | 1 | 0 |
| Comparator D | 0 | 0 | 0 | NA | 0 | 0 |
| Comparator E | 0 | 0.94 | 0 | 1 | NA | 0 |
| Comparator X | 0.09 | 1 | 1 | 1 | 1 | NA |
| **Random effects** | | | | | | |
| Comparator A | NA | 1 | 1 | 1 | 1 | 0.65 |
| Comparator B | 0 | NA | 0.03 | 0.99 | 0.18 | 0 |
| Comparator C | 0 | 0.97 | NA | 1 | 0.91 | 0 |
| Comparator D | 0 | 0.01 | 0 | NA | 0 | 0 |
| Comparator E | 0 | 0.82 | 0.09 | 1 | NA | 0 |
| Comparator X | 0.35 | 1 | 1 | 1 | 1 | NA |

**Abbreviations:** NA, not applicable

**Supplementary Table S6** Illustrative example: indirect comparison of intervention X versus comparator A

|  | **Estimate** | **95% LCL** | **95% UCL** | ***p*-value** | ***I*^2^** | ***Q* statistic** | ***p*(*Q*)** |
| --- | --- | --- | --- | --- | --- | --- | --- |
| **Intervention X vs comparator A (Bucher ITC)** | −1.48 | −22.33 | 19.38 | 0.8896 |  |  |  |
|  |  |  |  |  |  |  |  |
| **Comparator A vs comparator B (pooled result)** | 55.84 | 38.03 | 73.64 | <0.0001 | 62.21 | 5.29 | 0.0709 |
| Study 1 | 58.80 | 35.87 | 81.73 | <0.0001 |  |  |  |
| Study 2 | 35.70 | 11.98 | 59.42 | 0.0032 |  |  |  |
| Study 3 | 66.50 | 55.13 | 77.87 | <0.0001 |  |  |  |
|  |  |  |  |  |  |  |  |
| **Intervention X vs comparator B (pooled result)** | 54.36 | 43.50 | 65.22 | <0.0001 | 85.86 | 21.22 | <0.0001 |
| Study 4 | 61.40 | 55.91 | 66.89 | <0.0001 |  |  |  |
| Study 5 | 43.50 | 38.01 | 48.99 | <0.0001 |  |  |  |
| Study 6 | 59.80 | 38.24 | 81.36 | <0.0001 |  |  |  |
| Study 7 | 55.80 | 46.20 | 65.40 | <0.0001 |  |  |  |

**Abbreviations:** ITC, indirect treatment comparison; LCL, lower confidence limit; UCL, upper confidence limit

**Supplementary Figure 1** LSR model. *Abbreviations*: *LSR* least squares regression


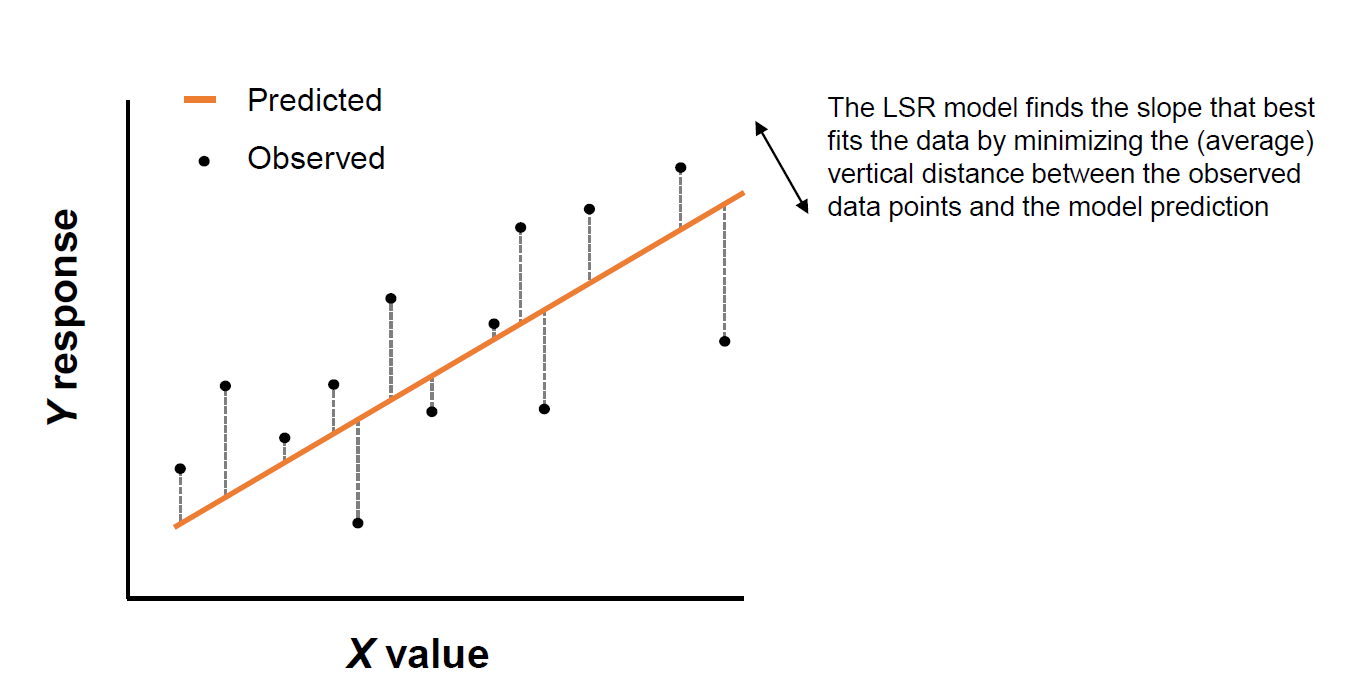


**References**

1. Pharmaceutical Benefits Advisory Committee (PBAC). Guidelines for preparing submissions to the Pharmaceutical Benefits Advisory Committee, version 5.0. 2016. <https://pbac.pbs.gov.au/content/information/files/pbac-guidelines-version-5.pdf>. Accessed 3 November 2023.

2. Institute for Quality and Efficiency in Health Care. Institute for Quality and Efficiency in Health Care. General Methods, version 7.0. 2023. <https://www.iqwig.de/en/about-us/methods/methods-paper/>. Accessed 20 November 2023.

3. Cochrane. Chapter 16.8.1: Bayesian methods. Cochrane Handbook for Systematic Reviews of Interventions version 5.1. <https://handbook-5-1.cochrane.org/chapter_16/16_8_1_bayesian_methods.htm>. Accessed 13 March 2024.

4. Dias S, Caldwell DM. Network meta-analysis explained. Arch Dis Child Fetal Neonatal Ed. 2019;104:F8–F12.

5. Rosenberger KJ, Xing A, Murad MH, Chu H, Lin L. Prior choices of between-study heterogeneity in contemporary bayesian network meta-analyses: an empirical study. J Gen Intern Med. 2021;36:1049–57.

6. Thano AN. Evidence synthesis: from meta-analysis to network meta-analysis with an application in patients with COPD. Athens University of Economics and Business, Department of Statistics; 2017.

7. Rhodes KM, Turner RM, Higgins JP. Predictive distributions were developed for the extent of heterogeneity in meta-analyses of continuous outcome data. J Clin Epidemiol. 2015;68:52–60.

8. Ren S, Oakley JE, Stevens JW. Incorporating genuine prior information about between-study heterogeneity in random effects pairwise and network meta-analyses. Med Decis Making. 2018;38:531–42.

9. European Network for Health Technology Assessment (EUnetHTA). Individual Practical Guideline Document. D4.3.1 Direct and indirect comparisons. 2022. <https://www.eunethta.eu/wp-content/uploads/2022/12/EUnetHTA-21-D4.3.1-Direct-and-indirect-comparisons-v1.0.pdf>. Accessed 1 November 2023.

10. Coordination Group on Health Technology Assessment. Methodological guideline for quantitative evidence synthesis: direct and indirect comparisons. <https://health.ec.europa.eu/document/download/4ec8288e-6d15-49c5-a490-d8ad7748578f_en?filename=hta_methodological-guideline_direct-indirect-comparisons_en.pdf>. Accessed 18 September 2024.

11. Dias S, Welton NJ, Sutton AJ, Ades AE. NICE Decision Support Unit Technical Support Documents. In: NICE DSU Technical Support Document 2: a generalised linear modelling framework for pairwise and network meta-analysis of randomised controlled trials. London: National Institute for Health and Care Excellence (NICE); 2014.

12. Deeks JJ, Higgins JPT, Altman DG, editors. Chapter 10: Analysing data and undertaking meta-analyses. In: Higgins JPT, Thomas J, Chandler J, Cumpston M, Li T, Page MJ, Welch V, editors. Cochrane Handbook for Systematic Reviews of Interventions version 6.4 (updated August 2023). Cochrane; 2023. <https://training.cochrane.org/handbook/current/chapter-10#section-10-10-1>. Accessed 2 November 2023.
